# Effects of Ethanol on Sensory Inputs to the Medial Giant Interneurons of Crayfish

**DOI:** 10.3389/fphys.2018.00448

**Published:** 2018-04-27

**Authors:** Matthew E. Swierzbinski, Jens Herberholz

**Affiliations:** Neuroscience and Cognitive Science Program, Department of Psychology, University of Maryland, College Park, MD, United States

**Keywords:** alcohol, crayfish, neurons, muscimol, inhibition

## Abstract

Crayfish are capable of two rapid, escape reflexes that are mediated by two pairs of giant interneurons, the lateral giants (LG) and the medial giants (MG), which respond to threats presented to the abdomen or head and thorax, respectively. The LG has been the focus of study for many decades and the role of GABAergic inhibition on the escape circuit is well-described. More recently, we demonstrated that the LG circuit is sensitive to the acute effects of ethanol and this sensitivity is likely mediated by interactions between ethanol and the GABAergic system. The MG neurons, however, which receive multi-modal sensory inputs and are located in the brain, have been less studied despite their established importance during many naturally occurring behaviors. Using a combination of electrophysiological and neuropharmacological techniques, we report here that the MG neurons are sensitive to ethanol and experience an increase in amplitudes of post-synaptic potentials following ethanol exposure. Moreover, they are affected by GABAergic mechanisms: the facilitatory effect of acute EtOH can be suppressed by pretreatment with a GABA receptor agonist whereas the inhibitory effects resulting from a GABA agonist can be occluded by ethanol exposure. Together, our findings suggest intriguing neurocellular interactions between alcohol and the crayfish GABAergic system. These results enable further exploration of potentially conserved neurochemical mechanisms underlying the interactions between alcohol and neural circuitry that controls complex behaviors.

## Introduction

Alcohol is one of the most abused drugs worldwide with devastating impacts on health and economy. Despite its well-documented negative effects, research aimed at understanding the underlying neurobehavioral mechanisms has progressed slowly. Unlike other drugs of abuse, alcohol exposure produces biphasic behavioral responses, which are expressed by initial hyperexcitability followed by motor incoordination and sedation. In addition, alcohol exerts its cellular effects by interacting with multiple neurotransmitter system, namely serotonin (Barr et al., 2003; Wolf and Heberlein, 2003; Ferraz and Boerngen-Lacerda, 2008) and y-amino-butyric acid (GABA) (Mehta and Ticku, 1988; Mihic et al., 1997; Lobo and Harris, 2008; Kumar et al., 2009).

Given the complexity of alcohol’s interplay with neural function, more recent research efforts have focused on animal models that display easily quantifiable behaviors and nervous systems that contain fewer and more accessible neurons. Across invertebrate studies, the symptoms of ethanol (EtOH) intoxication are highly conserved. This includes research performed in nematodes (Topper et al., 2014), fruit flies (Lee et al., 2008), and crayfish (Friedman et al., 1988; Macmillan et al., 1991; Blundon and Bittner, 1992; Swierzbinski et al., 2017). Similar to vertebrates, lower doses produce disinhibition while higher doses produce increased incoordination and/or decreased activity. The nematode, *Caenorhabditis elegans*, has proven a powerful tool for investigating the cellular and molecular targets of addictive substances, including alcohol (Schafer, 2004; Mitchell et al., 2007; Topper et al., 2014). The fruit fly, *Drosophila melanogaster*, has been used to study the effects of alcohol on behavior after genetic manipulations, which provided new insights into the interactions between EtOH and neurobehavioral mechanisms (Kong et al., 2010; van der Linde and Lyons, 2011; Devineni and Heberlein, 2013; Robinson and Atkinson, 2013). Crayfish have been used to study the effects of EtOH on behavior and synaptic transmission at the neuromuscular junction. Friedman et al. (1988) demonstrated an increase in righting time (when crayfish were placed on their back) in newly intoxicated animals, but not in those that had been chronically exposed. This study also reported a dose-dependent effect, with lower concentrations of EtOH increasing, and higher concentrations reducing, transmitter release and synaptic potentials at the neuromuscular junction. In addition, crayfish have been used to study drugs of abuse other than alcohol. For example, Huber et al. (2011) demonstrated that crayfish display conditioned place preference following injections of cocaine and amphetamines, and morphine injections into the crayfish brain have been shown to facilitate locomotion and exploratory behaviors (Imeh-Nathaniel et al., 2014).

Previous results from our lab demonstrated that juvenile crayfish are behaviorally and physiologically sensitive to EtOH and that this sensitivity is affected by recent social experience (Swierzbinski et al., 2017). When placed in a water-filled tank that contained various concentrations of EtOH, crayfish became more intoxicated over time in a dose-dependent manner, and they progressed through distinct stages of behavioral change. This included increased locomotor activity displayed by spontaneous tail-flips in the absence of any threat, which was followed by decreased activity and incoordination when they fell on their backs, eventually unable to right themselves. Surprisingly, animals housed with conspecifics prior to EtOH exposure progressed through these stages of increasing intoxication more rapidly than animals that were socially isolated. Importantly, we were further able to demonstrate parallel effects of EtOH on single neurons using intracellular electrophysiology. We found that the excitability of the crayfish’s lateral giant (LG) escape neurons was facilitated by EtOH exposure and, similar to our observations in freely behaving animals, the amount of EtOH-induced facilitation was dependent on recent social experience. Lastly, we also found that removing brain-derived tonic GABAergic inhibition to the local LG circuit reduced the sensitivity of the LG neurons to EtOH exposure, which led us to hypothesize that EtOH interacts with GABA receptors in the LG circuit.

Our current work expands on this notion utilizing a different set of crayfish giant neurons, which are key components of the medial giant (MG) escape circuit. We decided to target the MG neurons for three main reasons: (1) To see if the effects of EtOH would generalize across different escape circuits in crayfish, and (2) To contribute to our understanding of the neurochemical mechanisms in a circuit that is currently understudied, and (3) To provide a first glimpse into the interplay between alcohol and GABAergic inhibition in a circuit that receives multi-modal sensory activation and is of behavioral relevance.

While the LG circuit is one of the best described neural circuits in the animal kingdom, the MG circuit is much less well understood (Edwards et al., 1999). Both the LG and the MG circuits are considered to be hardwired and to produce reflexive, stereotyped escape behaviors. If a predatory attack is directed to the rear, activation of the LG neurons is sufficient to initiate an escape response that pitches the animal upward and forward away from the stimulus. Conversely, a frontal attack will activate the MG neurons and elicit a behavioral sequence that thrusts the animal backward (Herberholz et al., 2004). The LGs and MGs have many features in common: They can both be activated by strong and phasic sensory inputs, and a single impulse is necessary and sufficient to coordinate the entire escape response. Both travel the length of the nervous system and share most of their motor and inhibitory elements, which are often connected by electrical synapses (Wine and Krasne, 1982). However, the circuits also exhibit some important differences, which led to an imbalance in research efforts. Most experiments have been devoted to the LG circuit, which receives mechanosensory inputs from innervated hairs and proprioceptors on the abdomen and tail as well as subthreshold modulatory inputs from rostral body areas (Liu and Herberholz, 2010). The physiological characteristics of the LG circuit can be studied in each of the six abdominal body segments, which are readily accessible, and can be isolated from the rest of the animal. Analysis of the MG neurons has progressed much slower. The MG neurons, a pair of cells that are electrically coupled to each other, have their cell bodies located in the supraesophageal ganglion (brain). Activation of one MG in the brain typically activates the other MG, resulting in a pair of action potentials that descend toward the abdomen. The MG axons project along the entire nerve cord where the descending action potentials activate motor neurons connected to flexor muscles in all abdominal segments (Wiersma, 1947; Wine and Krasne, 1972; Wine, 1984).

The role of inhibition has been studied intensively in the LG circuit, but is mostly unknown for the MG circuit. The LG neurons undergo both phasic and tonic GABAergic inhibition (Roberts, 1968; Vu and Krasne, 1993; Vu et al., 1997). Ambient GABA released “globally” by interneurons descending from the brain has prolonged, modulatory function in regulating LG neuron excitability. The synapses for tonic inhibition are located on the dendrites of the LG neurons, and the inhibitory effects are mediated by ligand-gated chloride channels (Vu and Krasne, 1993; Vu et al., 1993). Up- and down-regulation of tonic inhibition and corresponding neuronal threshold has been observed in a number of situations, e.g., during feeding or restraint (Krasne and Wine, 1975; Krasne and Lee, 1988). In addition, the LG neurons also rapidly inhibit themselves after they discharge. This phasic “recurrent” inhibition is thought to happen more proximally, near the spike initiation zone, which is located at the initial axon segment. The behavioral purpose of recurrent inhibition is to prevent subsequent activation of the neurons after producing a single tail-flip. Recurrent inhibition can prevent firing of the LG neurons regardless of the magnitude of excitatory inputs, whereas strong excitation can override tonic inhibition. Chloride-mediated inhibition in crayfish often causes neurons to depolarize due to an outflow of chloride ions. In the LG circuit, the opening of such chloride channels shunts the current that flows toward the LG spike initiation zone (Roberts, 1968; Edwards et al., 1999).

Unlike the LG neurons, which receive primarily mechanosensory excitatory inputs, sensory activation of the MG neurons has not been mapped out in detail. However, Glantz and Viancour (1983) showed that they receive excitatory inputs from the antenna I, the main olfactory organ, and the antenna II, the primary mechanosensory organ. The antenna II also contains a smaller number of bimodal chemotactile receptors in a number of crustaceans, but it is unknown whether these non-olfactory receptors provide any inputs to the MG neurons (Sandeman et al., 1992; Liu and Herberholz, 2010; Derby and Weissburg, 2014). Visual activation of the MG neurons has been confirmed behaviorally (Liden and Herberholz, 2008; Liden et al., 2010). For example, when juvenile crayfish were presented with a threatening visual stimulus while they were approaching a food odor release point, they either displayed a freezing response or an escape tail-flip. All tail-flips were mediated by activity in the MG neurons, which was confirmed by using a pair of bath electrodes located in the water to record the large field potentials generated by the MG neurons during the tail-flip (Liden and Herberholz, 2008). Moreover, when the food odor concentration was increased, MG-mediated tail-flips were suppressed as animals decided to freeze (and stay close to the food) rather than escape. This suggests that the response of the MG neurons to visual stimulation was modulated by olfactory signals (Liden et al., 2010). Internal state, such as hunger, also affected MG threshold in these experiments (Schadegg and Herberholz, 2017). Together, these behavioral experiments illustrated the sensitivity of the MG neurons to multi-modal sensory cues as well as intrinsic signals.

The MG circuit also plays a significant role during crayfish aggression (Edwards and Herberholz, 2005). The formation of a social dominance relationship between two crayfish includes escalating levels of aggression, and activation of the MG neurons is often observed during the decision point when the future dominants and subordinates are determined. A sharp transition in behavior typically identifies the loser of a fight, and MG-mediated tail-flips are used by the emerging subordinate to break off an escalated encounter (Herberholz et al., 2001). In response to attacks from a natural predator, the MG circuit is engaged more than other escape circuits (Herberholz et al., 2004), and much like the LG circuit, it is also susceptible to other strong and phasic mechanosensory stimuli (Herberholz, 2009).

Given the known influences of alcohol on invertebrate behavior and the extensive background on escape circuitry in crayfish, the MG circuit presents a well-suited experimental model for testing the effects of EtOH on the function of identified neurons that are involved in a number of important behavioral outputs.

## Materials and Methods

Juvenile crayfish (*Procambarus clarkii*) were used for all electrophysiological experiments. Animals were purchased from Atchafalaya Biological and housed in large communal tanks (76 cm × 30 cm × 30 cm, L:W:H) with 50–100 crayfish until social isolation. Since social experience significantly affects alcohol sensitivity in crayfish, both behaviorally and physiologically (Swierzbinski et al., 2017), we decided to use only pre-isolated animals for the current study. All animals were socially isolated for 7–10 days in small individual tanks (15 cm × 8 cm × 10 cm, L:W:H) prior to the experiments. Approximately 2 cm of gravel covered the bottom of the isolation tanks, and each tank was oxygenated using air stones (BubbleMac Industries). Before social isolation, animals were checked for intactness (no signs of any major bodily injury), and only animals that had not recently molted (within 48 h) were used. On the day of social isolation, crayfish were given one single shrimp pellet (Aqua Pets Americas). Before surgical procedure, each animal was measured from their rostrum to their telson (tail-fan). The average body length of all crayfish used in the experiments was 3.5 ± 0.21 cm (*N* = 35). Each animal was only used once.

### Surgery and Electrophysiology

**FIGURE 1:**
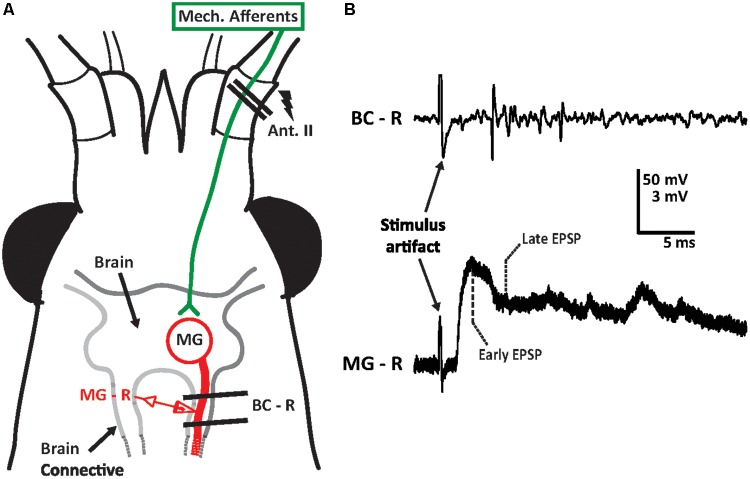
Medial giant (MG) circuit diagram for antenna II inputs and sample recording trace. **(A)** Crayfish head depicted with location of the antenna II. Sensory inputs from other sensory systems are not shown. Supraesophageal ganglion (brain) of the crayfish was surgically exposed from the ventral surface. Extracellular hook electrodes (two parallel black lines) were used to electrically stimulate the sensory afferents of the antenna II nerve (lightning bolt) and to record evoked activity in the ipsilateral brain connective (BC-R). Sharp intracellular glass electrodes were inserted into the MG neuron in the brain connective ipsilateral to the stimulated antenna II nerve to record MG neuron activity (MG–R). The size of the crayfish nervous system is not to scale. **(B)** A sample recording trace of the brain connective (BC–R) and MG neuron (MG–R) in response to electrical stimulation of the antenna II nerve (at 3 V). Extracellular activity (top trace) as well as MG’s early (3 ms after stimulus artifact) and late (6 ms after stimulus artifact) post-synaptic potentials (bottom traces) are shown.

Animals were chilled on ice for 15 min and pinned down ventral side up in a Sylgard-lined dish filled with 40 ml of crayfish saline. Pins were inserted into the telson (tail-fan) and thorax to secure the animal in place. Ventral cuticle was removed from the abdomen in order to expose the ventral nerve cord (VNC) of the abdomen and cut all motor roots of the abdominal ganglia in order to reduce movements induced by activation of the MG neurons. Cuticle rostral to the mandibles was removed and the green glands were extracted to expose the brain connectives (BC) where the impalement of the MG neuron was performed (**Figure 1A**). The MG was impaled using sharp micropipette electrodes pulled (Sutter Micropipette Puller; Sutter Instruments) from glass capillary tubes (World Precision Instruments; outer diameter: 1 mm, inner diameter: 0.58 mm). Intracellular electrodes were backfilled with 2 M potassium acetate and had resistances between 20 and 35 MΩ. The antennal II nerve was exposed by removing a rectangular piece of cuticle from the basal segment of the antenna. An extracellular silver wire hook electrode (Teflon coated wire; AM-Systems; uncoated diameter 0.127 mm) was placed on the nerve. Contact with the antenna II nerve was verified through observation of spontaneous and tactile-evoked action potentials. Post-synaptic potentials (PSPs) in the MG neuron were elicited through electrical stimulation of the ipsilateral antenna II nerve using a Grass stimulator (Model S88). Stimulation of one antenna II nerve almost never led to an action potential in MG, even at voltages just below direct (non-synaptic) stimulation of MG. However, post-synaptic potentials of several millivolts in amplitude could be reliably evoked. Intracellular signals were amplified using a microelectrode amplifier (Axoclamp 900A, Molecular Devices). Extracellular recordings were amplified using an A-M Systems differential amplifier (Model 1700) and digitized using a Digidata 1440A (Molecular Devices). The stimulating voltage was increased from 0 V until a sizable PSP could be observed, then increased until additional voltage produced no further change in the PSP. The voltage was then decreased to a voltage roughly at the midpoint between these two values. An inter-stimulus interval (ISI) of 90 s was used for all experiments. All experiments were conducted in a grounded Faraday cage.

Medial giant PSP amplitudes were analyzed at several time intervals after the start of the experiments. For each time point, two measurements were made (**Figure 1B**): the PSP amplitude at 3 ms following the stimulus artifact (termed “early”) and the PSP amplitude at 6 ms after the stimulus artifact (termed “late”). This analysis was based on previous experiments in the LG circuit; here, the early peak of the PSP reflects a mostly excitatory component, while the later time point is associated with postexcitatory inhibition, or a combination of excitatory and inhibitory inputs. Sweeps recorded during the saline baseline (∼10 min) were averaged for each animal and early and late component amplitudes were recorded. For each experiment, averaged PSP values recorded during baseline, experimental, and washout phases were normalized to the baseline average from all animals. Therefore, values above 100% of baseline demonstrate an increase over the averaged starting amplitudes, while values below 100% of baseline demonstrate a decrease. Electrophysiological data was recorded and stored using Molecular Devices pClamp 10 software. Data analysis was performed using Clampfit.

### Pharmacology

Solutions were introduced to crayfish preparations through a gravity-flow superfusion system consisting of glass reservoirs of solutions placed on top of the Faraday cage. The flow rate of the superfusion was held constant at 5 ml/min using a Baxter flow control device (Baxter International Inc.). This flow rate was checked before each experiment. Excess solution was removed from the dish through the use of a peristaltic pump (Thermo-Scientific FH100). All crayfish preparations were immersed in a modified van Harreveld’s solution (van Harreveld, 1936), a standard crayfish saline consisting of the following salts (in concentrations in mM): 202 NaCl, 5.37 KCl, 13.53 CaCl_2_, 2.6 MgCl_2_, and 2.4 HEPES (Antonsen et al., 2005; Liu and Herberholz, 2010). Saline was also used as a vehicle to deliver pharmacological agents to the preparations.

### Experiment Procedures

#### Experiment 1 (MG PSP Changes Over Time)

Previously, repeated stimulation of the LG while superfused with normal crayfish saline produced weak sensitization in some preparations (Swierzbinski et al., 2017). To measure how MG PSPs are affected during long-term repeated stimulations while superfused with normal crayfish, preparations were exposed to a 10 min saline baseline followed by 90 min of normal crayfish saline.

#### Experiment 2 (Alcohol Effects on MG PSP)

We have previously demonstrated the sensitivity of the LG circuit to 100 mM EtOH. To see if EtOH’s effects generalize across tail-flip circuits, preparations were exposed to baseline, then 60 min of 100 mM EtOH (4.6 g of ethyl alcohol solution mixed in 1 L of saline solution), and finally 60 min of washout with normal crayfish saline.

#### Experiment 3 (GABAergic Pharmacology of MG)

To further explore the presence of GABAergic inhibition in the MG circuit, preparations were exposed to the GABA_A_ antagonist, picrotoxin or the GABA agonist, muscimol. These preparations received 10 min of saline superfusion (baseline), followed by 30 min of drug exposure (25 μM PTX or 25 μM muscimol), and finally 60 min of saline (washout). In a subgroup of muscimol-exposed preparations (*N* = 3), the MG neuron was impaled with two intracellular electrodes to measure the change in input resistance during muscimol exposure. The input resistance of the MG was measured through injection of positive and negative currents (-40 to 40 nA) for 30 ms using the second intracellular electrode placed in the MG neuron in close proximity to the recording electrode. Voltage changes in MG membrane potential caused by current injections were recorded. In between injections, the ipsilateral antenna was electrically stimulated to produce MG PSPs to obtain recordings of both input resistances and post-synaptic potentials before (baseline), during muscimol application, and after (washout).

#### Experiment 4 (Interactions Between Muscimol and EtOH)

To test the interactions of muscimol and EtOH, preparations were exposed to 25 μM muscimol before being exposed to 100 mM EtOH. Preparations were given 10 min saline (baseline), 30 min of muscimol exposure, followed by 30 min of EtOH exposure, and finally 60 min of normal saline (washout).

### Statistical Analysis

All data is presented as means ± SEM except for animal sizes where standard deviation is shown. Statistical tests were performed using IBM SPSS (Version 23). Since some of our data failed normality as determined by Shapiro–Wilk Test, we used non-parametric tests throughout. We used Friedman as our omnibus test followed by pairwise comparison with Wilcoxon Signed Rank Test. We did not apply Bonferroni adjustment because our comparison across multiple levels (e.g., time points) would have likely resulted in a type II error. Statistical results are reported in the text and indicated in the figures.

## Results

### MG Action Potentials and PSPs Change Over Time

**FIGURE 2:**
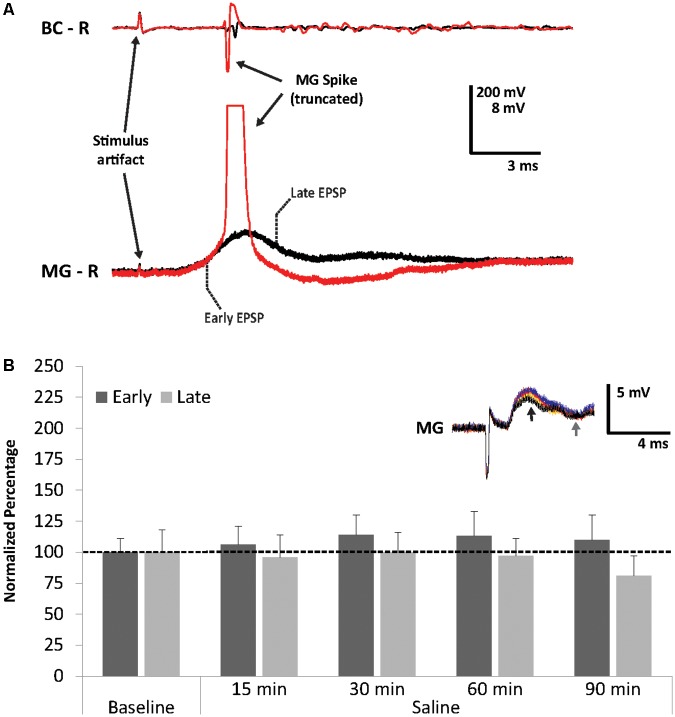
Superthreshold nerve shock and effects of repeated sensory stimulation on early and late MG PSPs. **(A)** MG action potential in response to antenna II nerve stimulation. **(B)** Average percent of PSP baseline values during 90 min of saline superfusion of the MG neuron (*N* = 5) and repeated stimulation (ISI = 90 s) of the antenna II nerve. Means ± SEM are presented. *Inset*: Example of a single MG PSP recording; black trace = baseline, orange trace = 30 min, red trace = 60 min, blue trace = 90 min. Arrows indicate early (black) and late (gray) PSPs.

We found that antenna II nerve stimulation reliably produces MG PSPs, but it generally fails to evoke an action potential in MG. Increasing the stimulus voltage will increase MG PSP amplitudes to a maximum level that is below spiking threshold. Increasing voltage further can fire MG directly (i.e., non-synaptically). Although we used voltage levels below the maximum in our experiments, in a few cases MG fired in response to antenna II stimulation when the appropriate stimulus voltage was determined. **Figure 2A** shows one such example. The MG spike rises from the early part of the PSP (3.5 ms after the stimulus artifact) suggesting that this part of the PSP consists of mostly excitatory synaptic inputs following antenna II stimulation.

Since our previous work had shown that the crayfish LG neurons become modestly sensitized after repeated sensory stimulation in normal crayfish saline (Swierzbinski et al., 2017), we first tested the effect of repeated stimulation on MG neuron excitability. After 10 min of baseline recordings, preparations (*N* = 5; 3.6 ± 0.16 cm) were perfused for 90 min with fresh crayfish saline and the antenna II nerve was stimulated every 90 s. We observed minor fluctuations in PSP amplitudes in both early and late PSP components throughout this time period (**Figure 2B**). Although PSPs changed slightly compared to baseline level at 15 min (Early = 106.2 ± 15.3%, Late = 96.0 ± 17.7%), 30 min (Early = 113.9 ± 16.2%, Late = 99.8 ± 15.5%), 60 min (Early = 112.5 ± 20.1%, Late = 96.9 ± 14.4%), and 90 min (Early = 110.0 ± 20.3%, Late = 80.7 ± 16.2%), none of these changes were significantly different from average baseline values (Friedman Tests; Early: Chi-Square = 1.914, df = 5, *p* = 0.861; Late: Chi-Square = 2.829, df = 5, *p* = 0.726). This is similar to what we observed in our earlier study of the LG circuit (Swierzbinski et al., 2017), and confirms that no major changes occur in these preparations over the course of 90 min of continuous stimulation and recordings.

### Alcohol Effects on MG PSP

**FIGURE 3:**
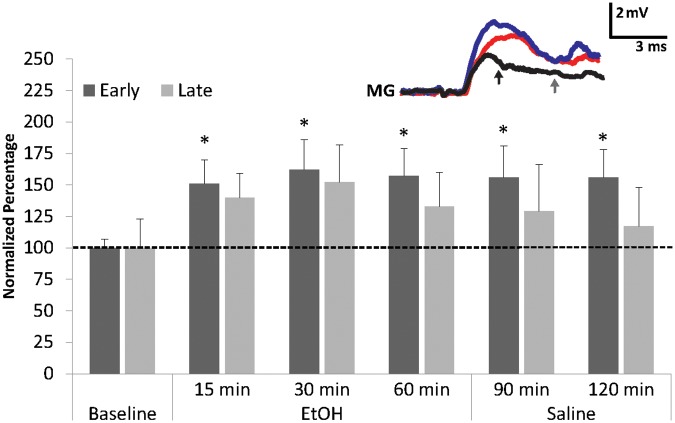
Effects of EtOH application on early and late MG PSPs. MG preparations (*N* = 8) were exposed to 10 min of saline baseline (Baseline), 60 min of 100 mM EtOH (EtOH), and finally 60 min of washout with normal saline (Saline). ^∗^ indicates values that were significantly different (*p* < 0.05) from baseline. Means ± SEM are presented. *Inset*: Example of a single MG PSP recording; black trace = baseline, red trace = 60 min, blue trace = 120 min. Arrows indicate early (black) and late (gray) PSPs. A Gaussian low-pass filter was applied to reduce electrical noise in the recording.

To investigate the effect of EtOH on the MG neuron, preparations (*N* = 8; 3.48 ± 0.23 cm) were exposed to 100 mM EtOH, a concentration found to be effective in socially isolated crayfish LG preparations (Swierzbinski et al., 2017). Similar to the LG, we found that EtOH exposure increased both early and late MG PSP amplitudes (**Figure 3**). The increases from baseline were significant (Friedman Test: Chi-Square = 12.929, df = 5, *p* = 0.024) for the early component after 15 min (150.6 ± 18.8%, Wilcoxon Signed Rank Test; *z* = -2.521, *p* = 0.012), 30 min (162.1 ± 24.3%, Wilcoxon Signed Rank Test; *z* = -2.380, *p* = 0.017), and 60 min (156.9 ± 21.9%, Wilcoxon Signed Rank Test; *z* = -2.240; *p* = 0.025). For the late PSPs, the responses to EtOH (15 min: 140.5 ± 19.3%; 30 min: 151.6 ± 30.3%; 60 min: 133.5 ± 27.3%) were not significant (Friedman Test; Chi-Square = 8.768, df = 5, *p* = 0.118).

The early MG PSP was resistant to saline washout. Average amplitudes of the early PSP recorded after 30 min (156.0 ± 25.0%; Wilcoxon Signed Rank Test: *z* = -1.960, *p* = 0.05) and 60 min (156.1 ± 22.5%; Wilcoxon Signed Rank Test; *z* = -2.380, *p* = 0.017) of washout remained significantly higher than average baseline level. Late PSP, however, decreased during washout from levels recorded during EtOH exposure and measured 128.8 ± 36.6% (30 min) and 116.9.3 ± 30.9% (60 min); these amplitudes were not significantly different from baseline level. The result suggests that EtOH has a facilitatory effect on the early MG PSP and a weaker non-significant effect on the late PSP. This is similar to what was observed in the LG circuit where EtOH made LG more excitable and increased its firing rate (Swierzbinski et al., 2017).

### GABAergic Pharmacology of MG

The LG is known to be inhibited through GABAergic mechanisms (Edwards, 1990; Vu and Krasne, 1992; Vu et al., 1997), but little is known regarding the MG. We next explored the role of GABAergic effects by measuring the effects of a GABA receptor antagonist (picrotoxin; PTX) and agonist (muscimol) on MG PSPs evoked through electrical stimulation of antenna II afferents.

**FIGURE 4:**
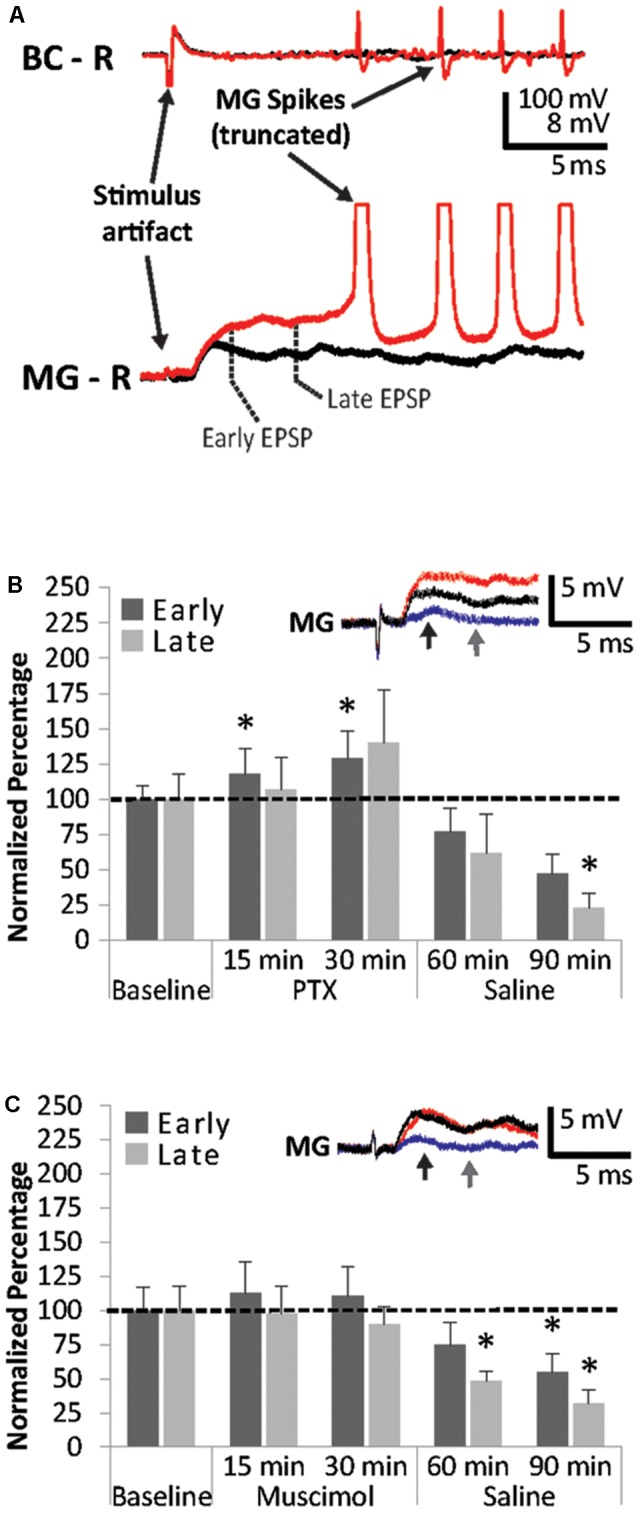
Effects of PTX and muscimol applications on early and late MG PSPs. **(A)** Superfusion of high concentrations of PTX cause repeated MG spikes rising from the late PSP. **(B)** MG preparations (*N* = 5) were exposed to 10 min of saline baseline (Baseline), 30 min of 25 μM picrotoxin (PTX), and 60 min of washout with normal saline (Saline). Early and late PSPs are compared to their average baseline values. **(C)** MG preparations (*N* = 8) were exposed to 10 min of saline baseline (Baseline), 30 min of 25 μM muscimol (Muscimol), and 60 min of washout with normal saline (Saline). Early and late PSPs are compared to their baseline values. ^∗^ indicates values that were significantly different (*p* < 0.05) from baseline. Means ± SEM are presented. *Inset*: Example of a single MG PSP recording; black trace = baseline, red trace = 30 min, blue trace = 90 min. Arrows indicate early (black) and late (gray) PSPs.

Higher concentrations of PTX (>25 μM) often produced convulsions in the pinned-down preparation and MG spikes rising from the later PSP components. An example is shown in **Figure 4A**. Although we used 25 μM PTX to avoid evoking these spikes in subsequent experiments, the observation suggests that the later MG PSP component consists of PTX-sensitive inhibition. Since excitatory inputs likely contribute to the late PSP as well, this excitation is released from inhibition following PTX application (Vu et al., 1997). Because we rarely observed spikes less than 8 ms after the stimulus artifact during PTX treatment, this supports our notion that MG PSPs are similar to LG PSPs where an early, mostly excitatory PSP is followed by a later component, which is dominated primarily by inhibition (Roberts, 1968).

However, both early and late MG PSP components experienced significant changes when preparations (*N* = 5; 3.38 ± 0.31 cm) were exposed to 25 μM PTX and during washout (Friedman Tests; Early: Chi-Square = 16.800, df = 4; *p* = 0.002; Late: Chi-Square = 14.720, df = 4; *p* = 0.005). The early PSP was significantly facilitated at 15 min (117.5 ± 17.6; Wilcoxon Signed Rank Test: *z* = -2.023, *p* = 0.043) and 30 min (128.5 ± 19.6; Wilcoxon Signed Rank Test: *z* = -2.023, *p* = 0.043) of PTX treatment compared to average baseline (**Figure 4B**). Early PSP amplitudes did not change significantly during washout at 30 min (76.5 ± 16.9%; Wilcoxon Signed Rank Test: *z* = -1.753, *p* = 0.08) and 60 min (47.3 ± 14%; Wilcoxon Signed Rank Test: *z* = -1.753, *p* = 0.08). Late PSPs were not significantly affected by PTX treatment at 15 min (106.9 ± 22.8; Wilcoxon Signed Rank Test: *z* = -0.405, *p* = 0.686) and 30 min (139.7 ± 37.6; Wilcoxon Signed Rank Test: *z* = -1.483, *p* = 0.138). After 30 min of saline washout, late PSP amplitudes changed compared to baseline (61.9 ± 27.6%; *z* = -1.483, *p* = 0.138) and were significantly lower than baseline after 60 min of saline washout (22.9 ± 10.5%; Wilcoxon Signed Rank Test: *z* = -2.023, *p* = 0.043).

Surprisingly, muscimol, a known ionotropic GABA receptor agonist (Johnston, 2014), produced only small PSP amplitude changes in all preparations (*N* = 8; 3.45 ± 0.14 cm) during application, but significant suppression of both early and late PSPs during washout (Friedman Tests; Early: Chi-Square = 14.700, df = 4; *p* = 0.005; Late: Chi-Square = 10.100, df = 4; *p* = 0.039) similar to those observed in PTX (**Figure 4C**). Amplitudes measured after 15 min of exposure changed to 113.4 ± 22.8% (Wilcoxon Signed Rank Test: *z* = -0.420, *p* = 0.674) and 97.9 ± 19.7% (Wilcoxon Signed Rank Test: *z* = -0.840, *p* = 0.401) for the early and late MG PSP, respectively. After 30 min of muscimol exposure, little additional change was observed for the early PSP (110.8 ± 21.4%; Wilcoxon Signed Rank Test: *z* = -0.420, *p* = 0.674) and late PSP (90.4 ± 12.5%; Wilcoxon Signed Rank Test: *z* = -0.700, *p* = 0.484). During washout, the early PSP was reduced to 74. 5 ± 16.0% at 30 min (Wilcoxon Signed Rank Test: *z* = -1.820, *p* = 0.069) and 55.3 ± 13.3% at 60 min, the latter being a significant decrease from baseline (Wilcoxon Signed Rank Test: *z* = -2.240, *p* = 0.025). The late PSP was reduced significantly at both the 30 min (47.7 ± 7.4%; Wilcoxon Signed Rank Test: *z* = -2.240, *p* = 0.025) and the 60 min (31.7 ± 9.8%) time points (Wilcoxon Signed Rank Test: *z* = -2.100, *p* = 0.036).

To determine whether a relationship exists between muscimol application and MG input resistance, possibly indicating an effect of muscimol on MG itself, we measured MG input resistance in a subset of these preparations (*N* = 3; 3.5 ± 0.17 cm). The average input resistance was 75 ± 21.4 KΩ, which is comparable to an existing report of MG input resistance measured in adult crayfish (Glantz and Viancour, 1983). In parallel to small changes in PSP amplitudes over the course of 30 min of muscimol exposure in both the early (93.7 ± 49.3%) and the late (97.2 ± 21.9%) PSPs, the input resistance changed only marginally (107.1 ± 28.0%) compared to the input resistance measured during baseline. PSP amplitudes decreased after 60 min of saline compared to baseline (Early = 49.5 ± 27.6%; Late = 51.3 ± 25.5%), and input resistance of the MG neuron showed a parallel decline (81.8 ± 13.6%).

### Interactions Between Muscimol and EtOH

**FIGURE 5:**
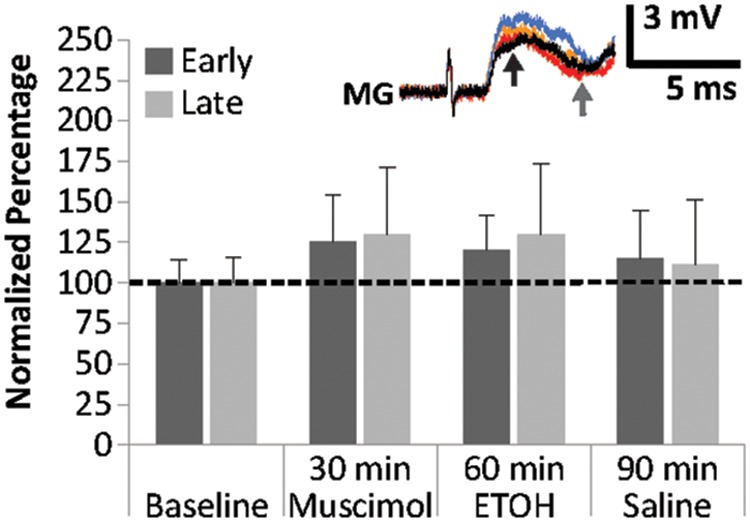
Effects of muscimol application prior to EtOH exposure on MG PSPs. Preparations (*N* = 6) were exposed to 10 min of saline baseline, 30 min of 25 μM muscimol, 30 min of 100 mM EtOH, and 60 min of saline washout. Means ± SEM are presented. *Inset*: Example of a single MG PSP recording; black trace = baseline, orange trace = 30 min, red trace = 60 min, blue trace = 90 min. Arrows indicate early (black) and late (gray) PSPs.

To explore the interactions between the GABA receptor agonist muscimol and EtOH on MG PSPs, preparations (*N* = 6; 3.6 ± 0.16 cm) were treated with 25 μM of muscimol for 30 min, then exposed to 100 mM EtOH for another 30 min, and finally to saline for 60 min (**Figure 5**). Muscimol exposure produced only small changes in early and late MG PSPs compared to baseline. Early PSP amplitude values measured 114.4 ± 20.9% (15 min) and 124.9 ± 29.6% (30 min), and late PSPs measured 117.5 ± 24.5% (15 min) and 129.8 ± 41.7% (30 min). After EtOH was added to the preparations, both the early PSP amplitude (15 min: 124.5 ± 25.1%; 30 min: 119.5 ± 21.8%) and the late PSP amplitude (15 min: 140.1 ± 39.9%, 30 min: 129.7 ± 43.8%) exhibited only minor changes. In addition, saline washout produced only minor effects in early PSPs (30 min: 114.8 ± 29.7%, 60 min: 125.1 ± 37.9%) and late PSPs (30 min: 110.5 ± 40.5%, 60 min: 130.0 ± 52.2%). None of the changes in early or late MG PSP were statistically significant from average baseline (Friedman Tests; Early: Chi-Square = 3.000, df = 6; *p* = 0.809; Late: Chi-Square = 4.214, df = 6; *p* = 0.648). Two important conclusions can be drawn from this experiment: Pre-exposure to muscimol prior to EtOH application suppresses the facilitating effect of EtOH compared to when EtOH is applied alone, and EtOH exposure after muscimol eliminated the reduction of PSP amplitudes typically seen during muscimol washout.

## Discussion

The cellular workings underlying the complex interplay between alcohol and nervous system function are still poorly understood. The crayfish present a highly suitable model to probe into the neurocellular and neurochemical mechanisms, and it allows linking drug-induced changes in neural activity to whole animal behavior. We have previously reported that crayfish are behaviorally sensitive to EtOH exposure and progress through quantifiable, discrete stages of intoxication (Swierzbinski et al., 2017). Behavioral sensitivity to EtOH is dependent on social history of the individual, which we demonstrated by showing that communally housed crayfish respond to EtOH more quickly than socially isolated conspecifics. Lastly, we were able to determine that behavioral effects evoked by EtOH in crayfish are paralleled on the level of single neurons, the LG interneurons.

In our current study, we expanded on these prior findings. We focused our work on a different escape circuit, the MG, because we wanted to know if the effects of EtOH would generalize across neural circuits in crayfish. We selected the MG circuit due to its higher complexity compared to LG, which is illustrated by integration of multimodal sensory signals as well as superior relevance for behaviors such as predator escape, aggression, decision-making and risk-taking (Herberholz et al., 2001, 2004; Liden et al., 2010). Although our current study focused on antenna II stimulation of MG, this work can be expanded by including other sensory modalities (e.g., visual) in the future (Liu and Herberholz, 2010).

We found that MG neurons respond to sensory afferent stimulation in similar fashion compared to LG neurons (Liu and Herberholz, 2010). The evoked compound post-synaptic potential (PSP) is of mostly biphasic shape (**Figure 1**), separated by a downward deflection, which has been attributed to post-excitatory inhibition in LG (Vu et al., 1997). Unlike LG, however, the MG is nearly impossible to activate in a restrained preparation with sensory nerve stimulation alone. Previous work has shown that coincident inputs from the contralateral MG and subthreshold antenna II inputs can bring MG to threshold (Herberholz and Edwards, 2005). In addition, when subthreshold antenna II inputs are combined with superfusion of 500 μM serotonin, MG action potentials can be evoked (Hu et al., 2014). In our current experiments, we observed MG spikes only on rare occasions when the baseline stimulus voltages were determined, and we found that the MG action potential rises from the early PSP (**Figure 2A**) similar to previous observations made on the LG neuron. Application of high concentrations of the GABA-receptor antagonist PTX also evoked MG spikes, which always originated from the later parts of the PSP (**Figure 4A**). This is likely due to MG spike activation via uninhibited excitation and has been shown to occur in the LG (Vu et al., 1997). In LG, excitatory mechanosensory interneurons (e.g., Interneuron C) produce bursts of action potentials that contribute to the late PSP (e.g., Zucker et al., 1971). The occurrence of multiple MG spikes indicates that proximal recurrent inhibition has been eliminated by PTX, similar to what has been described for LG (Roberts, 1968). While these observations may suggest that the early MG PSP component is primarily of excitatory nature and the later part comprised of (mostly) inhibitory and excitatory inputs (similar to LG PSPs), additional work is necessary to fully characterize the contributions of antenna II synaptic inputs to the MG PSPs.

When we exposed preparations to 100 mM EtOH, the alcohol concentration was higher than those that produce effects in humans (e.g., the legal blood alcohol driving limit in the United States is ∼17 mM), but in line with other alcohol literature. In fact, one controversy of alcohol research (including rodent work) is the perplexing result that EtOH concentrations within the “normal biological range” are often insufficient to evoke neurophysiological responses (Aguayo et al., 2002). Recently, progress has been made to understand the mechanisms underlying this reduced sensitivity, which seem to be related, in part, to the GABA receptor subunits (Cui and Koob, 2017). We have shown previously that the LG neurons of socially experienced crayfish respond to lower EtOH concentrations (10–20 mM) compared to socially isolated animals (20–100 mM), which were used in the current study.

Exposure of the preparations to EtOH produced significant increases in the early MG PSPs (**Figure 3**). Although late PSPs increased as well, the changes were not statistically significant. Thus, EtOH facilitated early sensory inputs to the MG neuron, which is similar to LG where excitability in response to synaptic inputs from tail afferents was significantly enhanced by EtOH (Swierzbinski et al., 2017). We found that EtOH-induced facilitation was resilient to wash out (with saline) for the early MG PSPs, suggesting strong binding affinity of EtOH to post-synaptic receptors or sustained facilitation of synaptic inputs that produce the early PSP component.

It is unclear at this point how EtOH causes the increase in early MG PSP. Both pre- and post-synaptic effects are possible as well as interactions with multiple neurotransmitter systems. Our initial attempt focused on the role of the GABAergic system since the interplay between alcohol and GABA has been described in numerous publications and investigated in a large number of animal systems (e.g., Koob et al., 1998; Davies, 2003). In addition, GABAergic inhibition, including tonic inhibition, of the LG neurons is well established (Vu and Krasne, 1993; Vu et al., 1993; Edwards et al., 1999), and its role in regulating behavior has been documented (Krasne and Wine, 1975; Krasne and Lee, 1988). However, until now inhibitory mechanisms of the MG circuit other than those related to motor outputs have not been studied.

Application of a non-competitive GABA receptor antagonist (picrotoxin; PTX) resulted in larger PSPs (**Figure 4B**). The result was only significant for the early component suggesting that the early PSP is more affected by PTX than the late PSP. The effect is likely due to reduced GABAergic inhibition; however, since PTX is generally assumed to block the channel pore and prevent flow of chloride ions, it could also partially be attributed to an overall increase in MG input resistance. In addition, PTX interactions with invertebrate GABA receptors are more complex than in vertebrate systems where it reliably blocks ionotropic GABA receptors although more effectively for GABA_A_ receptors than GABA_C_ (also known as GABA_Arho_) receptors (Bormann and Feigenspan, 1995; Wang et al., 1995). PTX has been shown to block crustacean non-GABA mediated chloride channels (Albert et al., 1986), and crayfish interneurons vary in their response to PTX, some being highly sensitive and others being unaffected (Sherff and Mulloney, 1996; Miyata et al., 1997). This prior work suggested that at least some GABA-gated channels in crayfish have low affinity for PTX or are entirely non-sensitive to the antagonist.

A similar result to our finding (i.e., increase of early PSP) has been described by Vu and Krasne (1993) for the LG neuron. In intact preparations that contained the entire nervous system, PTX caused an increase in the early excitatory component (i.e., β-component) of the LG PSP evoked with tail afferent inputs. However, when the brain was separated from the tail (the location of the LG neurons) by sucrose gap or transection of the ventral nerve cord, PTX had no effect on the β-component. These differences were explained by changes in tonic inhibition of the LG, which originates in the crayfish brain and projects to the abdomen via descending interneurons (Vu and Krasne, 1993; Vu et al., 1993). Our work on the MG was done in restrained intact preparations where tonic inhibition was likely to occur; it is therefore possible that the PTX-induced increase in early PSP is related to suppression of tonic inhibition.

Tonic inhibition is widely distributed across the mammalian brain (e.g., Farrant and Nusser, 2005; Kullmann et al., 2005), produced by low levels of extracellular GABA, and mediated by slow-desensitizing ionotropic GABA receptors of distinct subunit composition, which can be blocked by PTX (Yeung et al., 2003; Wei et al., 2004). Interestingly, those receptors are also highly sensitive to alcohol (Sundstrom-Poromaa et al., 2002; Paul, 2006; Smith and Gong, 2007) and are considered a major cellular target for the drug in the mammalian nervous system (Valenzuela and Jotty, 2015).

In our own earlier work (Swierzbinski et al., 2017), we found that LG neurons of crayfish tail preparations (i.e., without tonic inhibition) were less excited by EtOH than semi-intact preparations (i.e., with tonic inhibition). This indicated that EtOH might interact with brain-derived tonic inhibition. The fact that lower concentrations of EtOH were required to activate the tonically inhibited LG than the disinhibited LG suggested that EtOH blocks tonic inhibition. Together with our current findings, one – of several – possibilities is that EtOH binds competitively to the receptors mediating tonic inhibition. EtOH has previously been shown to promote *or* prevent GABA receptor activation by the natural ligand in different mammalian neurons. EtOH-mediated *inhibition* of GABA receptor activity is widely known for the GABA_C_ receptor (Mihic and Harris, 1996; Yamakura and Harris, 2000; Borghese et al., 2016). Interestingly, it has been suggested that (some) invertebrate GABA receptors are similar to this receptor type, in part because they both are insensitive to the antagonist bicuculline (Swensen et al., 2000). Moreover, GABA_C_-like receptors have been identified in cultured thoracic neurons of lobster (Jackel et al., 1994), and cloning and expression of a GABA receptor subunit from the X-organ of crayfish revealed a homomeric, bicuculline-insensitive receptor similar to the vertebrate GABA_C_ receptor (Jiménez-Vázquez et al., 2016). However, no invertebrate GABA receptor has yet been determined to be fully homologous to the vertebrate GABA_C_ receptor (Martínez-Delgado et al., 2010). Importantly, GABA_C_ receptors are known to mediate tonic inhibition in the retina of mammals and possibly other areas of the brain as well (Jones and Palmer, 2009).

Taken together, one mechanism by which EtOH could facilitate early MG PSPs is by interfering with GABA activation of GABA_C_-like receptors located on the MG, and likewise, on the LG. These receptors could be located intra- or extra-synaptically and respond to phasic or tonic inhibition. However, this is only one possible scenario and several others must be considered. For example, EtOH could exert its effects presynaptically by facilitating transmitter release from sensory pathways that stimulate MG, or it could interact with post-synaptic receptors other than GABA. The purpose of our current paper was not to solve these questions. They provide, however, a useful concept and exciting avenues for additional experimentation.

Washout of PTX caused reduction of MG PSP amplitudes compared to baseline in our experiments, which was significant for the late PSP amplitude, indicating onset of strong inhibition after the GABA receptor blocker was removed. At this point, the mechanisms underlying this “rebounding” inhibition are unclear. It seems possible that GABA accumulates over time due to continuous tonic release and after repeated stimulation, and once the channel blocker is removed, strong GABA-mediated inhibition follows. Importantly, the aforementioned GABA_C_ receptor fails to desensitize even with maintained GABA (or other agonist) application, and PTX has lower efficacy for blocking this receptor compared to the GABA_A_ receptor when expressed in *Xenopus* oocytes (Wang et al., 1995; Chang and Weiss, 1999). Since GABA is cleared rapidly (i.e., within a few hundred microseconds) from the synaptic cleft in mammalian neurons (Nusser et al., 2001), build-up of tonically released GABA (and/or diffusion/spillover from phasic release) and subsequent activation of inhibitory receptors might be a more plausible explanation.

While we expected muscimol (an agonist for ionotropic GABA receptors) to reduce MG PSP amplitudes, it actually produced no statistically significant effect during exposure (**Figure 4C**). This is not in agreement with some reports in the literature showing that muscimol is capable of agonizing certain GABA receptors in crayfish (Hori et al., 1978; Krause et al., 1981; El Manira and Clarac, 1991). However, no prior experiments exist that measured the effects of muscimol on the MG circuit, and the types of GABA receptors present in this circuit are unknown. In mammals, muscimol competitively agonizes the GABA_A_ receptor, but only acts as a partial agonist on the GABA_C_ receptor. Thus, it can occupy the binding site and reduce the receptor’s response to the natural ligand, basically acting as an antagonist (Johnston, 2014). As mentioned earlier, it has been recognized that (at least some) crustacean GABA receptors, including those in crayfish, are indeed structurally and functionally similar to the vertebrate GABA_C_ receptor.

Along those lines, a GABA receptor subunit sequenced from crayfish (*P. clarkii*) showed high sequence similarity to a GABA receptor subunit previously cloned from lobster (*Homarus americanus*) and expressed in human embryonic kidney cells (Hollins and McClintock, 2000). Interestingly, for both the crayfish and lobster GABA receptor, pharmacology revealed that PTX blocked receptor currents, but bicuculline did not, and muscimol was much less effective as an agonist than GABA itself. Using American Lobster and Jonah crab, Northcutt et al. (2016) recently applied deep sequencing of transcriptomes and identified orthologs of two GABA_B_ type subunits as well as three GABA_A_ type subunits with similarity to *Drosophila* RDL, LCCH3, and GRD receptors. The sequence of the RDL-like receptor identified in their study was most similar to the crayfish and lobster GABA receptors described earlier. Importantly, the *Drosophila* RDL receptor also experiences lower binding affinity to muscimol than GABA (Buckingham et al., 1994), is insensitive to bicuculline, and shares other similarities with the vertebrate GABA_C_ receptor (Hosie et al., 1997).

During saline washout of muscimol, we found that PSP amplitudes decreased significantly for both the early and late PSPs compared to baseline levels. Since muscimol is expected to compete with GABA at the receptor binding site, this result may suggest that removal of muscimol during washout clears the binding sites for ambient GABA that has accumulated due to either ongoing tonic release, or is present during stimulus-evoked synaptic release. This effect could be potentiated since muscimol also acts as a weak GABA uptake inhibitor (Johnston, 2014). Although the described hypothesis is mostly speculative at this point, it is supported by prior work showing that GABA receptors exposed to long-term bath application of muscimol have high affinity for the natural ligand (i.e., GABA) after washout (Chang et al., 2002).

When we tested MG’s input resistance during and after muscimol exposure, we found no major changes. A minor increase with muscimol exposure was followed by a minor decrease during washout. Our sample size was small (*N* = 3), and the result should be interpreted with caution. Nonetheless, it hints at the possibility that the effects of muscimol are post-synaptic rather than presynaptic, and they are related to muscimol acting as a competitive partial agonist during exposure (as described for GABA_C_ receptors), which is then displaced by GABA during washout. Alternatively, tonic presynaptic effects could lead to prolonged transmitter release and increase in MG input resistance. A clearer picture would emerge if GABA currents could be measured in MG and more localized drug application was used, which might be possible in the future. Those additional experiments are needed to unambiguously identify post- and/or pre-synaptic mechanisms.

The suppression of PSP amplitudes for both PTX- and muscimol-treated animals and the decrease in MG input resistance during washout may also suggest “run-down” of the preparations at these late experimental stages. However, in our other experiments (e.g., saline; **Figure 2B**), the PSPs showed no sign of reduction for long-lasting experiments, indicating healthy preparations. Moreover, resting membrane potentials did not change (aside from small fluctuations) over the course of the experiments. Thus, we believe these effects to be based on GABA receptor interactions during washout of the drugs.

When we exposed our preparations to muscimol *before* EtOH was applied, we made two interesting and possibly interconnected observations (**Figure 5**): First, the facilitating effect of EtOH on the early MG PSP was eliminated after muscimol pre-treatment. Second, the significant decrease in MG PSP during washout of muscimol was muted when EtOH (instead of saline) was added to the preparations.

This may indicate that the addition of EtOH after muscimol produced an effect similar to saline washout of muscimol, and thus the normally observed increase of PSP amplitudes after EtOH application was counterbalanced by parallel suppression of MG PSPs via GABAergic inhibition. Although EtOH would possibly compete with GABA for receptor occupancy once the binding sites become available, EtOH can either activate or inhibit GABA receptors in mammalian neurons (e.g., Lobo and Harris, 2008). Thus, the stimulating effects of EtOH on the sensory-evoked MG PSPs could be in part mediated by its interaction with GABA (i.e., inhibition of GABA receptors), or produced independently. Conversely, it seems possible that GABAergic inhibition that normally follows muscimol washout and produces a reduction in MG PSP amplitudes has been counterbalanced by the stimulating effects of EtOH, either by EtOH-GABA interactions, via independent routes, or a combination thereof.

It is well known that EtOH affects other neurotransmitter systems, including serotonin (Barr et al., 2003; Ferraz and Boerngen-Lacerda, 2008). In crayfish, serotonin modulates the excitability of the LG circuit (Glanzman and Krasne, 1983; Teshiba et al., 2001), and this modulation is dependent on the social status of the animal (Yeh et al., 1996). Although our recordings were made in the MG neurons, the results have revealed some similarities between the two neural circuits, and thus serotonergic modulation of MG excitability may be expected. EtOH has also been shown to interact with the dopaminergic system in both invertebrates and vertebrates (e.g., Brodie et al., 1990; Yoshimoto et al., 1992; Bainton et al., 2000). More work is needed to further identify the neurocellular and pharmacological interactions between alcohol and crayfish neurotransmitters systems. Having identified two neural circuits that are accessible for single cell electrophysiology, and are both modulated by EtOH in a similar fashion, opens up new opportunities.

In summary, we found that EtOH increases post-synaptic potentials evoked by sensory inputs from the antenna II to the MG neuron. Although we only have indirect evidence that these early inputs are excitatory, it suggests that the stimulating effects of EtOH generalize across crayfish giant circuits. We also found that muscimol, a GABA receptor agonist, blocks the EtOH-induced facilitation of early post-synaptic potentials, which suggests interactions between EtOH and the GABAergic system, although they may not be direct. This is further supported by the lack of inhibition normally observed during muscimol washout with saline when saline is being replaced with EtOH. A possible role for tonic inhibition and GABA_C_ -like receptors is discussed in this context and provides an exciting framework that warrants further investigation.

## Ethics Statement

The study was exempt from ethical approval procedures because invertebrate animals were used.

## Author Contributions

JH and MS designed the study, contributed to data analysis, and wrote the manuscript. MS performed the experiments.

## Conflict of Interest Statement

The authors declare that the research was conducted in the absence of any commercial or financial relationships that could be construed as a potential conflict of interest.
